# A Distributed Sensor Network for the Control of a Bioclimatic House in Spain

**DOI:** 10.3390/s91008197

**Published:** 2009-10-19

**Authors:** Álvaro Gutiérrez, Javier Jiménez-Leube, Luis Magdalena

**Affiliations:** 1 ETSI Telecomunicación, Universidad Politécnica de Madrid, Avd. Complutense 30, 28040 Madrid, Spain; E-Mail: jleube@etsit.upm.es; 2 European Centre for Soft Computing, C. Gonzalo Gutiérrez Quirós S/N, 33600 Asturias, Spain; E-Mail: luis.magdalena@softcomputing.es

**Keywords:** smart house, energy control, bioclimatic house

## Abstract

The XXI century home should be a digital habitat, a connected residence, but at the same time it should be involved in sustainability and the environment. The location of new technologies at home, and its acceptance by the user, requires, among other actions, a significant diffusion and activity to be undertaken. This work proposes the development of a Smart House network designed for its integration into a sustainable and bioclimatic solar house. The paper focuses on a specific aspect of the house design, the control system bus, developed for the management of the different parameters, variables, sensors and actuators which coexist at home. Finally, the system has been installed in a laboratory bioclimatic house. Environmental variable experiments based on the control of passive elements, such as phase shift gels, are presented. Experimental results show that the system is able to benefit from the bioclimatic elements in the house by taking into account the user preferences.

## 1. Introduction

### 1.1. Motivation

For a long time, architecture has been based on logic, and therefore substantiated in climate criteria [1]. Traditional solutions make use of locally available resources which offer thermal inertia to the buildings thanks to the mass of cladding and partitions [2]. The use of thick ceramic, stone or soil walls give rise to magnificent results characterized by a thermal stability and a decrease energy dependence. However, this solution limits the energy accumulation capacity, because the materials could never go below the minimum temperature at night or above the maximum temperature during the day [3]. Furthermore, the heating or cooling of solids concentrates their energy only in the outer layers. Another drawback is that the energy load and unload takes place at a variable temperature. Hence they will never work at constant comfort conditions. Moreover, current construction methods have a tendency towards the lightness of the materials to cheap the structure and obtain a larger living area, which increase the looses on thermal inertia systems.

On the other hand, the bioclimatic architecture [1, 3–5] represents the use of materials and constructive elements under sustainability criteria. It represents the optimum-energy generation concept by means of the active or passive accumulation [6], distribution of renewable energies [7] and the integration of ecological building elements [8]. The bioclimatic architecture comes back to the common sense criteria, making use of technological elements, which give rise to results similar to the traditional ones, without loosing the benefits of the current constructive methods. Typical accumulation systems are substituted by others based on the heat exploitation stored in Phase Change Materials (PCMs) [9]. PCMs obtain energy from the outside and store it in form of energy represented as their liquid or solid state. As is known, the PCMs always obtain/release heat at the same temperature (e.g. 0 °C for the water). Therefore, by making use of chemical components to tune the changing temperature, new building designs may include PCMs to make use of their thermal capabilities. However, in real domestic implementations, the use of control systems becomes necessary to manage the air flows which allow the PCMs to change phase.

Because of new advances in home automation [10–12], there is an important field which is created when connecting bioclimatic and automation principles. At present, the smart house systems or *domotics* face a social change. Until very recently, domotics was described as a facility technical management. It was exclusively for the control of single devices in the residential or industrial sector, basically referring to isolated appliances, sensors or actuators. The new advances in technology, particularly in terms of information and communication technologies, has brought about a change in the approach in which the term *domotics* moves towards new concepts of digital habitat or connected environment [13]. Moreover, it evolves to concepts such as ubiquitous computing or ambient intelligence [14–16], which puts mankind in an environment which adapts to the needs and preferences of the user, at the same time it satisfies external conditions. Furthermore, it would be desirable that the low cost and energetic consumption sensor, actuator and control devices introduced by the user, were able to create a network which takes individual and collective decisions.

If we focus on specific details, the two more important elements are those related with the Personal Area Networks (PAN) and the control of sensors and actuators. Moreover, these elements must be integrated into the bioclimatic architecture and the renewable energy concept. Therefore, the XXI century home should be a digital habitat; a connected residence, but at the same time it should be involved in sustainability and the environment. The location of new technologies in the house, and its acceptance by the user, requires, among other actions, a significant diffusion and activity to be undertaken.

Following these lines of research, we pursue the implementation of a distributed sensor network for the control of bioclimatic and sustainable houses. In this work, we create and adapt a distributed network based on an industrial bus which confers the possibility of sensing environment variables and actuating different non-standard elements for the conditioning of the home. The present work focuses on specific aspects of the house design and the control system bus developed for the different parameters, variables, sensors and actuators systems that coexist within. The paper is organized as follows: The rest of Section 1 describes the house and offers a brief overview of bioclimatic and non-standard elements in it. In Section 2 the control bus and the nodes in the network are described. Section 3 sets out the user interface. This interface manages the orders given by the user to the house and monitors the status of the system thanks to its graphical interface. Experimental results are presented in Section 4. Finally, Section 5 concludes the paper and suggests future developments.

### 1.2. The Bioclimatic House

Nowadays, different research projects in Spain focus on the convergence of construction, bioclimatic and domotics systems [17]. However, for significant results, these studies should be carried out in environments with similar conditions to those in standard houses. Therefore, the system presented in this work has been installed in a solar bioclimatic house previously used for the *Solar Decathlon 2005* competition [18], organized by the Department of Energy of the United States of America. In this workshop, universities compete to design, build, and operate the most effective and energy-efficient solar-powered house. The project of the Universidad Politécnica de Madrid raised these aspects (i.e., technology, sustainability and diffusion) in a proposal for the creation of a prototype of a potential house for the XXI century. The aspects related with the technology and diffusion are reflected in the later application of the first prototype, which after having been in the *Solar Decathlon*, become a real laboratory and technology demonstrator in Spain.

**Figure 1.**
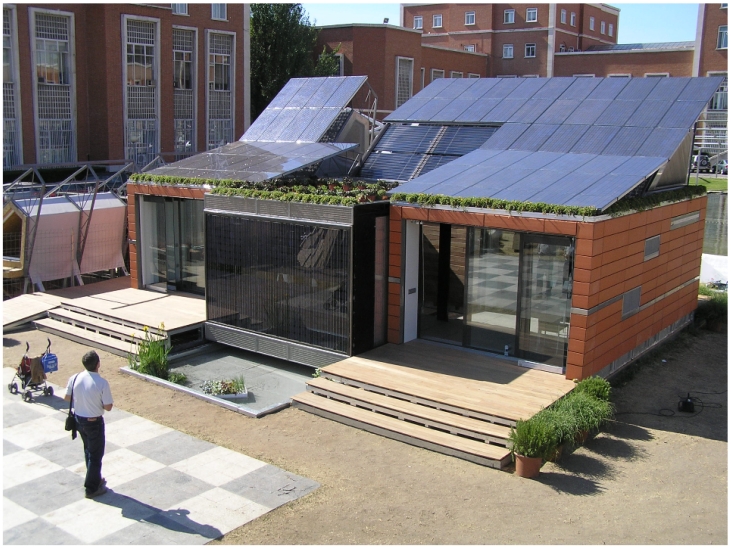

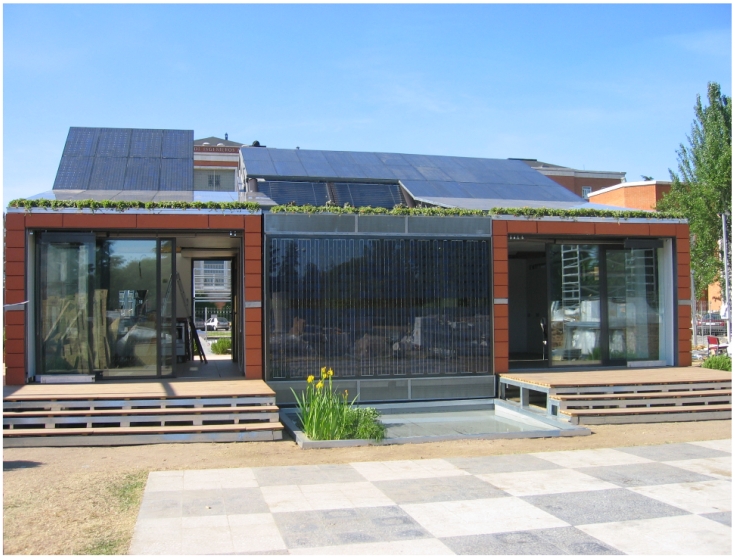
The bioclimatic solar house. (a) Bird's eye view. (b) South facade.

The house (see Figure 1) integrates sustainability elements based on the use of renewable energies, self-sufficiency energetic methods and recycled construction materials. The self-sufficiency is based on the correct use of the energy from a suitable control system, and the use of a bioclimatic design which reduces the energy needs for the achievement of adequate comfort levels inside the house.

The house has been designed to allow the air to circulate passively and create a comfortable environment without the need for complex elements. As previously mentioned, the classic storing procedures, used by the bioclimatic architecture, are those which accumulate heat or cold in the structure of the building. However, because of the dimensional limitations of the house, we have not used heavy elements in its construction. Hence, we have decided to use energy storage systems in the form of latent temperature; that is, to promote the phase change of a chemical substance storing the heat or cold in it.

**Figure 2.**
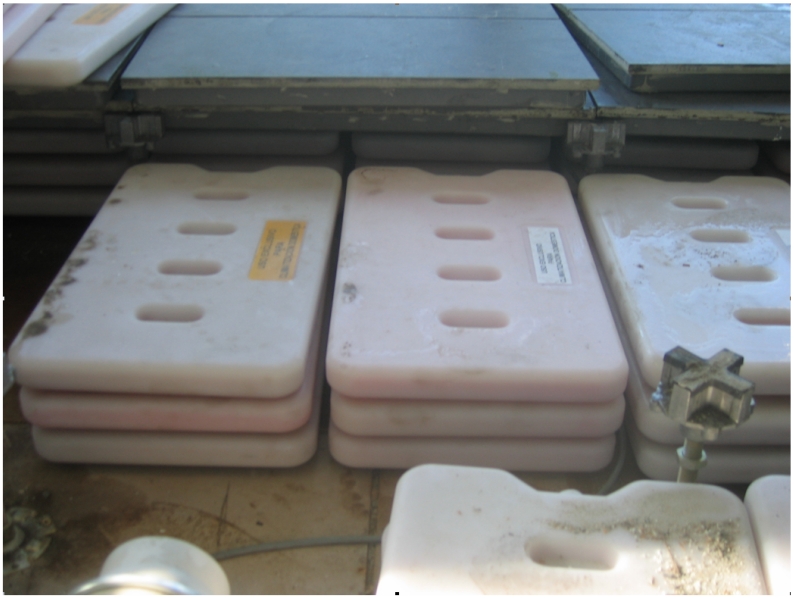

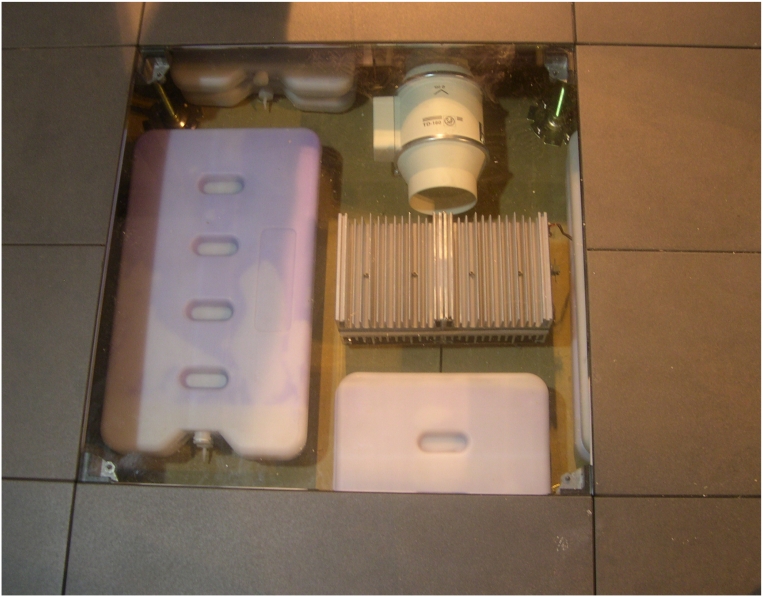
The false floor elements in the house.

The accumulation system used (i.e., Phase Sift Gel (PSG)) is made up of hermetic capsules of about 3 kg. each and of 28 × 48 × 3 *cm*^3^. These capsules are located under the floor on top of a thermal insulator which is supported by the house structure. There are four PSG layers, three of them in the form of capsules and the fourth one inside the ceramic pavement (see Figure 2a). Together with the PSGs we have included different active elements such as fans, peltiers and floodgates which, automatically controlled, create the conditions to modify the environmental variables in accordance with to the user needs. The system includes the following elements (see Figure 2b):

- **12 fans:** The fans are located in the false floor together with the PSGs. They allow the air movement which stores or obtains energy from the PSGs.
- **18 servomotors:** These are used for the grid and floodgate control. They modify the ventilation apertures in the house, redirecting it to the outside or the inside part of the house depending on the user needs.
- **6 peltiers:** The peltiers are used for the humidity control. When the humidity is higher than expected, the peltiers are activated and create a voltage potential which allows the air, when passing through them, to be dehumidificated.
- **150 PSG capsules:** These capsules include the already mentioned PSG which stores energy in terms of latent temperature.

The 12 fans, six in the east wing and six in the west wing, are located between the capsules and are in charge of the wind stream from which the energy is extracted. Each fan moves a flow of 160*m*^3^/*h*. Therefore, for the entire house a 1,920 *m*^3^/*h* air flow is achieved. Because the house has approximately 180 *m*^3^ volume, if all the fans are working for one hour, the system achieves 10.5 air renewals per hour. However, the number of active fans will change according to the air flow needs.

The environmental conditioning inside the house makes use of the PSGs, peltiers and grids. In winter, the energy used to acclimatize the house is solar radiation. It heats the PSG elements during the day, while they regulate the heat use during the night. On the other hand, in summer, the freshness during the night must be stored in the PSGs, and must be used to cool the house during the day. In any situation, the system must be able to share the energy stored to acclimatize the house for the whole day.

**Figure 3.**
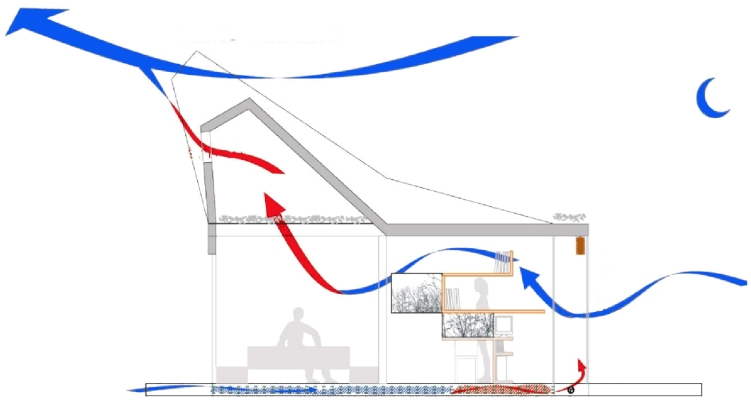

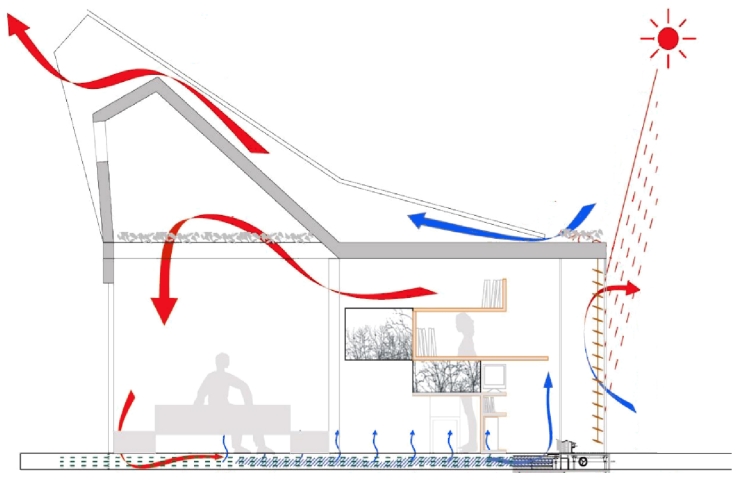
Temperature control in a summer (a) night and (b) day.

An important element of the system are the grid and floodgates. These elements are located in the ceiling and false floor of the north and south facades to modify the air flows from the indoors to the outdoors. For its correct working, the grids and floodgates must be positioned according to the needs of the house. For example, on a summer night (see Figure 3a), the north grids must be opened. Hence, the PSGs will obtain the freshness of the night while the rest of the house is aerated by opening the greenhouse and the north ceiling grids. When the day comes, the grids and greenhouse are closed outdoors (see Figure 3b). Therefore, the house must recirculate the temperature stored during the night, keeping the house fresh.

Furthermore, the humidity control is associated with the air streams generated by the existing fans. This air flow, when passing across the false floor, enters the dehumidification system (i.e., peltiers). The different peltiers are activated depending on the humidity and user needs.

## 2. The Control Bus

For the automatic working of all the elements presented in the previous section we have designed and implemented a distributed network which is able to measure all the environmental variables and activates the different fans, peltiers or servomotors. The sensor network intends for scalability. Therefore, any measurement variable could be inserted into the system by adding it to the network nodes. Flexibility is another key component of the network, where any node can be “hot-plugged” into it without the need to stop or modify the architecture.

The network has been developed based on the RS-485 bus [19]. This robust and industrially tested bus allows a fast reaction response to changes and future control implementations in the house. We decided not to use a specific commercial smart house system (i.e., EIB/KNX [20], lonworks [21] or X10 [22]) in favor of the industrial RS-485 bus for the following criteria:

- The house, in which the system is going to be installed, is not a typical house where commercial devices can be integrated. On the contrary, it is a laboratory where, from the very beginning, non-conventional devices are present: fans situated in the false floor, dehumidification systems, PSGs and laboratory instrumentation. This is because the house is nowadays used as a research environment where different technologies in the fields of energy, construction and domotics are studied. We hope in the short term we are able to transpose all these advances into commercial houses.
- The house itself is a very dynamic environment where different novel technologies coexists. The control of non-standard technologies implies the need for a fast reaction when they are tested. By using commercial technologies we are restricted to devices which exist in the market and some of them are not able to control specific elements in the house.
- Finally, we wanted to create a new device based on an already tested technology which makes the system robust and scalable, and which offers new control capabilities in the field of sustainability, bioclimatic and self-sufficiency.

The system designed holds up to 32 nodes without the need for a repeater, and a 1.5 km distance of transmission at 115,000 bauds. The implementation is based on a four-wire transmission, a physical-decentralized architecture and a logical-centralized architecture. The system has been designed by focusing on scalability and robustness. Following these principles, all the nodes (i.e., *NOD485*) are identical devices which allow different sensors and actuators to be connected. An embedded computer asks the nodes about the data measured and provides the information to the user. The network itself takes into account the user and house needs and triggers the different actuators according to predefined requirements. Although the system is based on a decentralized topology, the central computer stores all the data and control parameters that are accessible by the user from a web application (see Section 3). This communication allows the user to check the status of the house and to take the control of it, in case the algorithm does not fit his needs.

### 2.1. The Node

**Figure 4.**
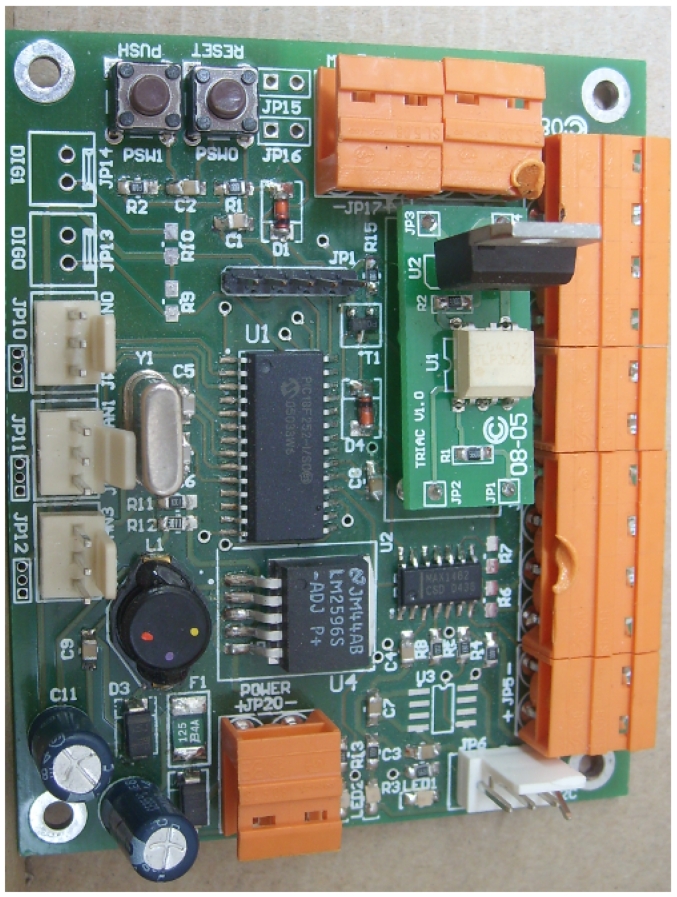

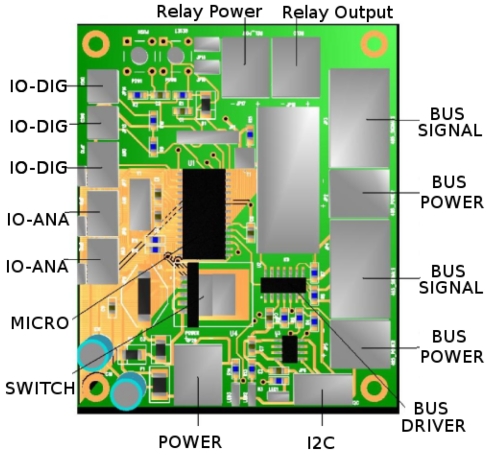
(a) The *NOD485*. (b) The functional architecture of the *NOD485*.

The *NOD485* (see Figure 4a) is an autonomous system controlled by its own microcontroller. The controller is in charge of communicating with the central computer, acquiring the information from the sensors attached and activating its different actuators. The board is equipped with two digital input/output lines, three analog inputs, one relay/triac output, an I2C bus and an RS-485 Full-Duplex bus (see Figure 4b).

The *NOD485* incorporates an isolated switched power supply. It allows a power input of 6–35 V and an efficiency of 85%–95% on the power conversion. It includes a safety power consumption system which disconnects the node if consumption exceeds 1.25 A. Once the safety system is engaged, the node short-circuits the RS-485 bus lines so as not to interfere with the other nodes in the network.

**Figure 5.**
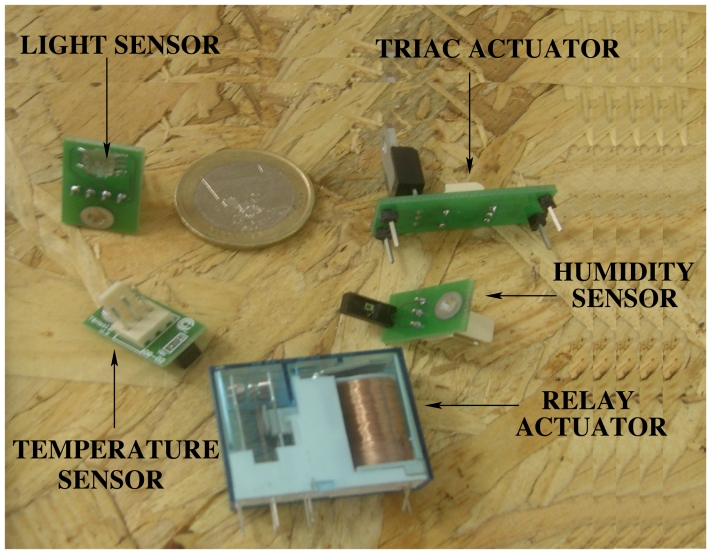
Sensor and actuator boards designed for its used on the *NOD485*.

The actual implementation of the node includes three physical variables to detect (i.e. temperature, humidity and light) and two actuators (i.e., a relay and a triac) (see Figure 5). The evolution of the solar bioclimatic house and the need for different functionalities will push on future sensor and actuator designs.

A monolithic sensor with on-chip signal conditioning has been chosen as the temperature sensor. It can be operated over the temperature range of 50 °C to +150 °C, making it ideal for its use in different locations. The signal conditioning eliminates the need for any trimming, buffering or linearization circuitry, greatly simplifying the system design and reducing the overall system cost. The output voltage is proportional to the temperature. The output swings from 0.25 V at 50 °C to +4.75 V at +150 °C using a single +5.0 V supply. Therefore, a simple linearization makes the device useful for our application:

(1) $$V_{\text{out}}=(V_{\text{cc}}/V_{\text{cal}})(1.375+\Delta V\;T_{a})$$

where *V_out_* is the voltage read at the analog to digital converter, *V_cc_* is the sensor power supply, *V_cal_* is the 5 V calibrated power supply, Δ*V* is the linearization steps of 22.5 mV/°C and *T_a_* is the temperature.

The humidity sensor used has a linear voltage output designed to face an analog to digital converter. The sensor is a laser trimmed thermoset polymer capacitive sensing element with on-chip integrated signal conditioning. The sensing element's multilayer construction provides resistance to application problems such as humidity, dust, dirt, oil, and common environmental chemicals. The sensor has a reduced current draw of 200 mA which makes it suitable for our application. Its relative humidity measurement is interpreted as follows:

(2) $$V_{\text{out}}=V_{\text{cc}}(0.0062RH_{\text{measure}}+0.16)$$

where *V_out_* is the voltage read at the analog to digital converter, *V_cc_* is the sensor power supply and *RH_measure_* is the relative humidity measurement.

However, the sensor is temperature dependent and a measurement correction must be included depending on the actual temperature:

(3) $$RH_{\text{real}}=RH_{\text{measure}}/(1.0546-0.00216T_{a})$$

where *T_a_* is in Celsius.

The light sensor used is a digital-output sensor with an I2C interface. It combines two photodiodes and a companding analog-to-digital converter on a single CMOS integrated circuit to provide light measurements over an effective 12-bit dynamic range with a response similar to that of the human eye. One of the photodiodes (channel 0) is sensitive to visible and infrared light, while the second photodiode (channel 1) is sensitive primarily to infrared light. An integrating ADC converts the photodiode currents to digital outputs. When a reading is achieved, the channel 1 output is subtracted from that of channel 0, compensating the effect of the IR light on the channel 0 photodiode. The result of this operation is the value obtained at the sensor output.

Both actuators, a relay and a triac, can be inserted into the *NOD485* using the same docking slot (see Figure 4b). The relay chosen is a miniaturized relay with an 8 A nominal current and 15 A peak current. It is able to control d.c. and a.c. motors of 230 V up to 3,000 W. However, when an a.c. motor is attached to the *NOD485*, there is a great possibility of coils linking between the relay and the motor. Therefore, we have design a specific extension board including a triac for the a.c. motor control. The triac selected is used both for an ON/OFF function or for phase control operation. It has been chosen because of its snubberless version. The snubberless network offers the suppression of an RC network and it is suitable for applications such as phase control and static switching on inductive or resistive loads.

### 2.2. Communication Protocol

**Figure 6.**
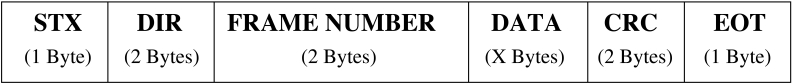
A communication frame structure.

The communication protocol is based on an ASCII implementation where control characters and value codifications coexist in a same frame. The frame design allows the definition of two non-printable characters at the beginning and end of the the frame, and an oversized error control system for future applications. The frame is defined as a variable structure represented in Figure 6 and described as follows:

- **STX (0×02):** It is the Start of Frame. This character establishes the starting point of a data frame.
- **DIR:** It is made up of 2 bytes which indicate the hexadecimal value of the node address in ASCII code. It is also used as the reception node address when the frame is sent by the computer. For example, if a frame is sent by the node “1D”, the field will include the 0×31 and 0×42 bytes which correspond to the “1” and “D” ASCII characters.
- **Frame Number:** 2 bytes which indicate hexadecimal value of the frame number. All the frames are numbered from 0×00 to 0×FF. When the frame number reaches its limit it is reset to the 0×00 value.
- **Data:** The amount of data sent in each frame is variable. It includes both special commands and data.
- **CRC-16:** 2 bytes used for the error detection transmission. The CRC generated corresponds to the whole frame not including the EOT and STX characters.
- **EOT (0×04):** It is the End of Frame character. This character establishes the end point of the data frame and indicates that the transmission has finished.

Any frame sent between two nodes or a single node and the computer is encapsulated according to the previous frame definition. Because any node can be reprogrammed remotely, the generation of new commands or specific data is a trivial task to be handled by the central computer. At present, four different data type groups have been created for communication with the temperature, humidity, light sensors and the actuators. Any data group has a question definition (about a specific value) and a standardized answer (returning the value asked). Table 1 shows the structure for each of these data. Finally, four specific commands have been defined as detailed in Table 2.

## 3. The User Interface

The system obtains a dynamic control, with a permanent communication with the user, allowing him to configure the system according to his needs. While the objective of the control software is to communicate with all the installed nodes and the central computer, the user interface is in charge of creating the instructions needed for the control system. Furthermore, the system architecture can be static, but the controllers developed on it may grow without limit. Because of the scalability and flexibility capabilities of the network, it can grow endlessly without the need for specific elements. Therefore, we have implemented a web application through which the user can insert all the data needed for the system definition. The user interface allows the user to install, program and modify the nodes available in the network. Moreover, it offers different visualization interfaces for the correct understanding of the user.

**Figure 7.**
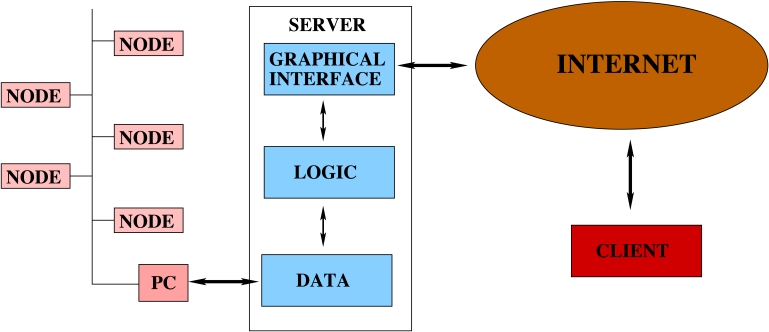
The software architecture.

The interface has been developed as a web service application based on application forms for the node, sensor and control algorithm configuration. It follows a client/server structure where the client is the process which asks for information and the server answers to the requests. It has been developed using a three-layered architecture: the first one takes care of the visualization based on HTML and CSS elements (graphical), the second one takes over the dynamic contents based on PHP code (logic) and the last one obtains data from or supplies it to the control system by means of XML files and a MySQL data base (data). Figure 7 shows the complete system architecture.

The interface offers the user the control of the system configuration and the actions to be carried out. The application provides a chained-form structure which facilitates the definition of the elements. Therefore, its main objective is to draw up a web application which serves as human-machine interface. In this way, it transmits all the instructions to the control system and it receives all the data for its monitoring.

**Figure 8.**
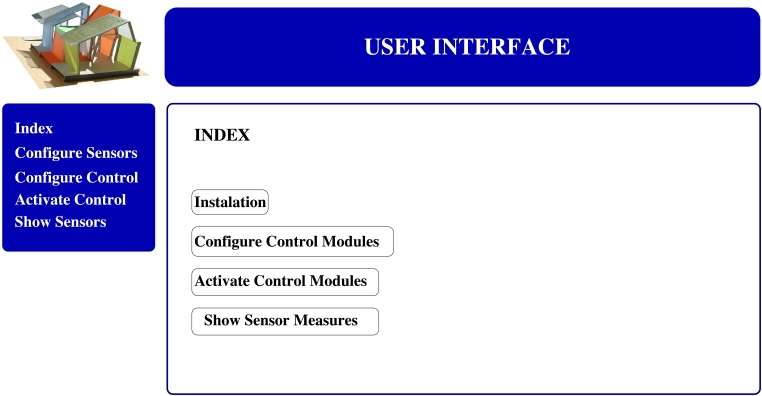

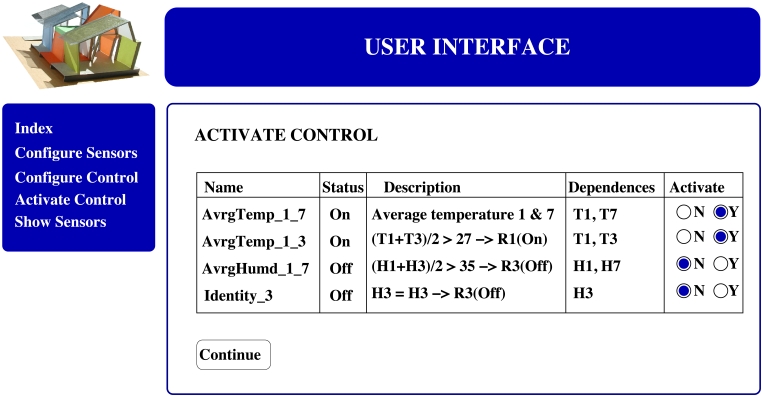
The user interface. (a) Main menu and (b) controller menu.

**Figure 9.**
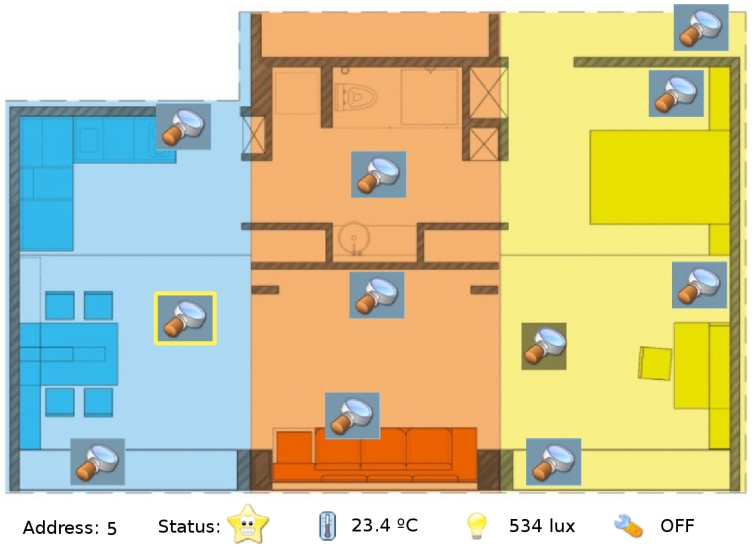

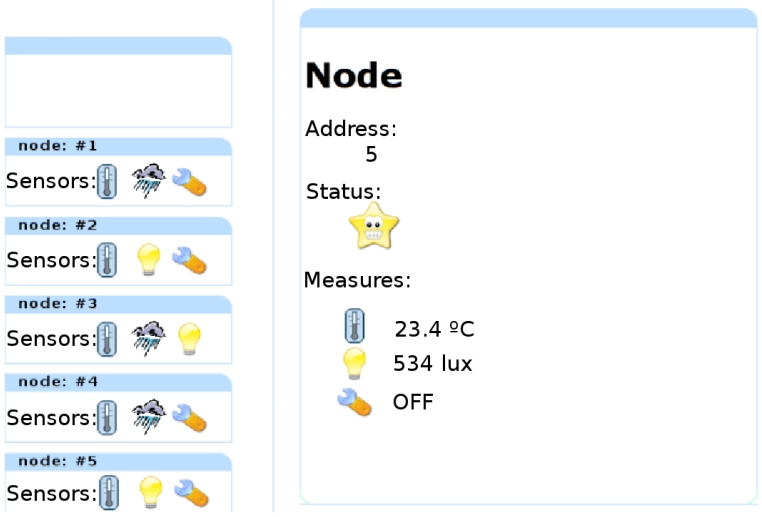
Data monitoring interface. (a) A map-like approach. (b) A node list approach.

The application is based on a main menu (see Figure 8a) which shows the user different options to communicate with the network:

- **Network configuration:** This module allows the user to add or remove new nodes in the network. It notifies the user about all the nodes detected into the system. The supplied list will include the previous installed nodes and new nodes physically introduced in the system.
- **Configuration of the controllers:** This is the core of the user interface and the most complex module. It allows the user to create or delete new controllers. The controllers are created based on logical terms. A simple and standard controller could be defined as follows: “If the average temperature measured by node 1 and node 3 is higher than 30°C and the temperature measured by node 5 is increasing then activate the air conditioner 2” which should be introduced into the system as “*if* ((*average*(*T*1(*t*), *T*3(*t*) > 30) & (*T*5(*t*) > *T*5(*t* − 1)) *then R*2(*ON*)”.
- **Activation of the controllers:** Different controllers created by different users can be available in the data base. However, not all the users will be interested in having all the controllers running at the same time. Hence, the interface allows the user to activate or deactivate the controllers according to his preferences (see Figure 8b).
- **Data monitoring:** The last module allows the user to visualize all the elements installed in the house. The information is plotted in two different configurations: *i)* A map-like approach (see Figure 9a) and *ii)* a node list approach (see Figure 9b).

## 4. Experimental Results

### 4.1. The Network in the House

**Figure 10.**
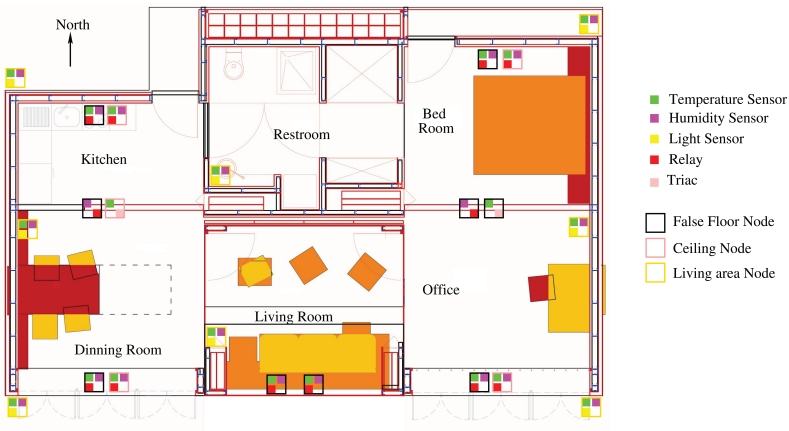
Actual location of nodes and their sensors in the house.

We have installed 22 nodes in the house according to the actual needs (see Figure 10). Four nodes (two on the north terrace and two on the south terrace) are located outdoors monitoring temperature, humidity and light conditions. Four nodes (one in the living room, one in the restroom, one on the east side and one on the west side) monitoring indoor temperature, humidity and light. Eight nodes are located on the north and south side of the house monitoring temperature and humidity in the ceiling and false floor and managing the grid automation with a relay actuator. Two nodes are located on the east and west false floor monitoring temperature and controlling six fans each for the air circulation thanks to the triac actuator. At the same location two more nodes monitor humidity and manage the activation of the peltiers with a relay actuator.

With all these nodes installed, we run two experiments. Both experiments try to control and optimize the temperature and humidity values, however the first experiment is considered a failed experiment because of the outdoor climate conditions as explained in Section 4.2. A second experiment shows the system working successfully, where the controllers try to counteract the outdoor conditions. Note that the experiments presented do not try to carry out an exhaustive study of environmental parameters. On the other hand, they are presented to show that the system architecture is working and that different controllers could be created and inserted into the system thanks to the user interface described in Section 3.

### 4.2. A Failed Experiment

The first experiment corresponds to the bioclimatic monitoring and control of the house from 10 am May 10th, 2009 to 5 pm May 12th, 2009. The main objective was to maintain the indoor temperature between 23 °C and 25 °C and the humidity at 30% managed by two simple controllers. The results obtained have not been as satisfactory as expected because of the climate conditions during the studied period and the week before. However, the measurements have allowed the control system to be validated.

**Figure 11.**
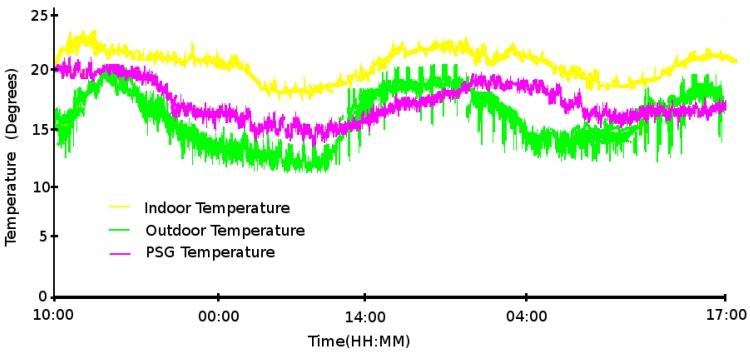

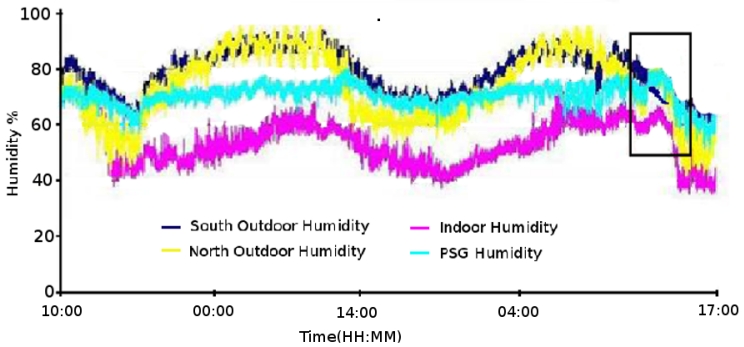
(a) Temperature and (b) humidity measurements for the first experiment period.

As shown in Figure 11a, none of the different areas of the house have reached the conditions planned. The grids and floodgates remained closed and the fans stopped during the whole experiment to keep the house as isolated as possible from the outdoor conditions. However, different conclusions can be extracted from the figure:

- The PSG temperature is more stable than the outdoor temperature. This is because of their thermal inertia.
- The cold (in our example) comes from outdoors and modifies the indoor conditions. We can observe a phase shift in the indoor temperature with respect to that of the outdoors. Moreover, the indoor temperature offers a more reduced amplitude because of the PSGs.
- For a successful climatization, the use of passive thermal conditioning is not enough when the outdoor conditions are not appropriate. An active element, such as a heat pump or air conditioning, is needed to help the energy storage on the PSGs. This is because, after a long period without the possibility of acquiring external energy, the system cannot reach the required values.

**Figure 12.**
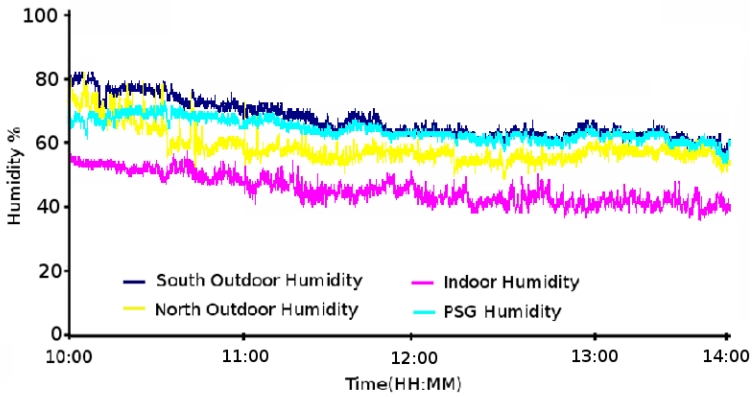

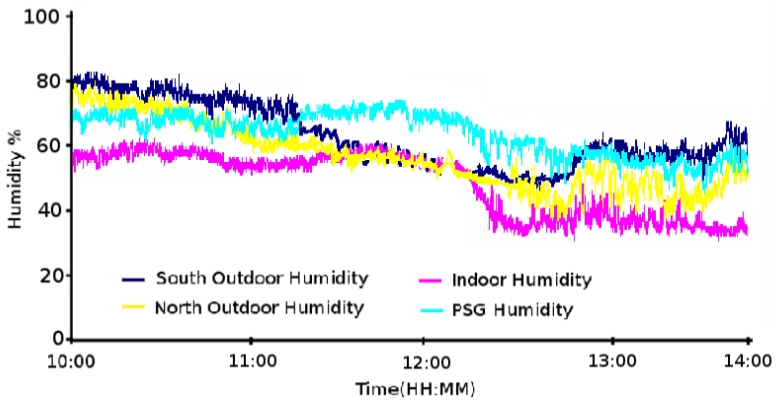
Humidity measurements (a) for a normal working day and (b) when the water pipe broke down.

If we look at the humidity measurements (see Figure 11b), we observe that the humidity has neither reached the intended value of 30%. However, a non-planned failure of the system made us realize about the correct working of the system. The problem was that a water pipe broke at noon on May 12th. This flood created an increase in the indoor humidity (see Figure 12b) contrary to the downward trend observed on the previous day (see Figure 12a). In a standard working, the system starts with a stored indoor humidity and gets reduced according to the influence of the outdoor humidity and the PSG saturation. On the other hand, when the failure occurred, the fans were fully activated together with the peltiers which tried to reduce the excess in humidity as observed in Figure 12b. Therefore, the humidity in the false floor (where the PSGs are located) gets increased.

### 4.3. A Successful Experiment

Because the first experiment climate conditions were not too satisfactory for testing the system, we ran a second experiment to demonstrate its correct working. For this experiment we sampled a working period from 10 a.m. May 21th, 2009 to 5 p.m. May 23th, 2009.

**Figure 13.**
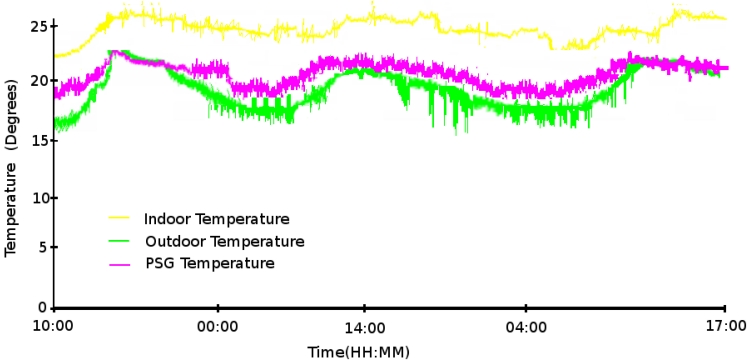

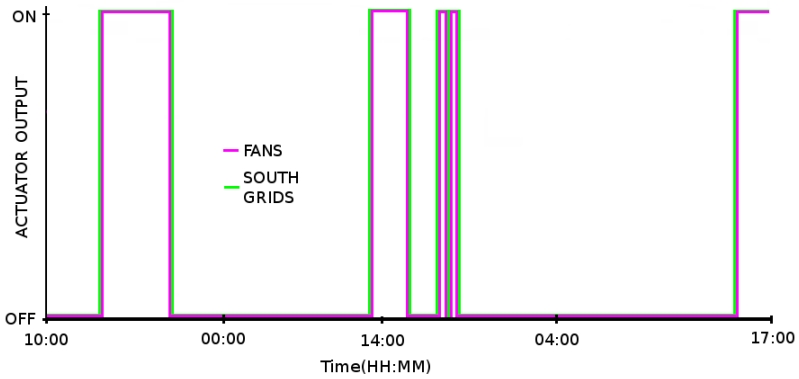
(a) Temperature and (b) actuator positions for the second experiment period.

Figure 13a, shows the indoor, outdoor and PSG temperature measurements for the complete experiment. The experiment is recorded after some sunny days, which explains that the indoor temperature starts around the optimal values. We observe, that as the day finishes, the outdoor temperature starts to decrease. This phenomenon influences the indoor and PSG temperature. Thanks to the thermal inertia of the PSGs, the system is able to keep the indoor temperature within an optimal interval in detriment to the PSG temperature.

Figure 13b shows the fans and south grids actuators. We observe that when the temperature is above 25 °C the south grids are opened and the fans activated. This situation makes the air circulate from the false floor (where the PSGs have a lower temperature) to cool the house. Once the optimal temperature (from 23 °C to 25 °C) has been reached, the fans are automatically stopped and the grids are closed. Hence, the house is closed to the outdoor and false floor thermal influences.

## 5. Conclusions

In this paper we have described a sensor network for the control of a bioclimatic solar house. The system creates a scalable and robust network, which allows the house to self-control different comfort variables according to the user needs. The system is based on the assumption that any domestic user creates a profile (controller) according to his needs or preferences on the temperature, humidity, light conditions, etc.

Although the solution adopted for the creation of the control bus, an RS-485 bus, seems to be a more tedious task than the use of commercial technology, we focus on the principle that it allows us to have a better control of the devices installed, and a faster reaction response to possible future changes independently of the existing solutions in the market. Following these ideas, as long as new devices are incorporated into the house new sensor/actuator extension boards will be added to our node.

A front-end application has been created for the user control. This application is based on web services, and allows the user to create, modify or activate different profiles according to his preferences. Moreover, a monitoring application which allows the user to observe the correct working of the system in any location, has been created.

Finally, the network has been installed and tested in a real prototype house used as a laboratory. This 60 m^2^ blueprint house offers the possibility of carrying out real and exhaustive tests as if they were in a standard house. The experiments carried out show that the system is working perfectly and is able to follow the user's orders. However, we have observed that the passive bioclimatic elements are not enough to maintain certain comfort variables when the outdoor climate conditions are not appropriate. Therefore, future work should be carried out on this direction.

Future work on the system will focus on the solar power control. Energy is an important aspect from the residential and overall perspective. Therefore a demand management which allows the user to estimate the best time to carry out some task is needed. For example, if a user needs to wash clothes, it could introduce the data into the system and the network should be able to offer the user the best time (within the limits already mentioned by the user) to start the washing machine. Therefore, the user could save energy and money because of the use of the solar energy locally generated. In a second step, we plan to automate all the household appliances. Hence, the user will supply the information on the appliance to use and the time interval, and the house should be able to allocate this information according to energy criteria.

## Figures and Tables

**Table 1.** Communication protocol: Data types defined.

Device	Function	Command	Description
Temperature	Question	T?	It asks a specific node for the temperature.
Temperature	Answer	T = XXX	The node replies with the temperature measured. The two first digits correspond to the degrees while the third one is the tenths.
Humidity	Question	H?	It asks a specific node for the humidity.
Humidity	Answer	H = XXX	The node replies with the humidity measured. The two first digits correspond to the ten and unit while the third one is the tenths.
Light	Question	L?	It asks a specific node for the light intensity.
Light	Answer	L = XXX	The node replies with the light intensity measured. The value is offered from 0 to 999 luxes.
Actuator	Order	R = X	The node switchs its value to open (X=1) or closed (X=0) and replies with an ACK command.

**Table 2.** Communication protocol: commands defined.

Command	Value	Description
ACK	0×06	It is sent when a frame has been received and decoded correctly.
NACK	0×15	It is sent when a frame has not been decoded correctly.
RESET	0×08	It forces the node to be reset. After this command, the node sends an ACK and resets itself. For the next 2 seconds the node enters a programing mode which allows the central computer to reprogram the device.
ALIFE	0×0C	This command is sent to a specific node to check if it is in working mode. If the board is correctly working, it answers with an ACK command.
